# List of reviewers

**DOI:** 10.1192/bjo.2021.11

**Published:** 2021-02-09

On behalf of the editorial board, the Editor-in-Chief would like to thank the following for their contribution as peer reviewers in the period from January 2020 – December 2020. Their anonymous work for the journal forms the foundation to its success, and is highly appreciated.

Joelle Abi-Rached
Samrachana Adhikari
Frances Adiukwu
Khalid Afzal
Tamas Agh
Jessica Agnew-Blais
Niruj Agrawal
Muhammad Muhsin Ahmad Zahari
Laura Akers
Umberto Albert
Regi Alexander
Laura Allison
Kelly Allott
Mandhar Almaqbali
Jaber Alqahtani
Daniel Alvarez-Fischer
Joanna Anderson
Kirstie Anderson
Gerard Anmella
Paul Appelbaum
S.M. Yasir Arafat
Sidse Arnfred
Elson Asevedo
David Attali
Raj Attavar
Agnes Ayton
Enrique Baca-Garcia
Alison Baird
Christian Bakker
David Baldwin
Matteo Balestrieri
Tali Ball
Samprit Banerjee
Yoram Barak
Lucy Barker
Helen Barratt
Darryl Bassett
Markus Bassler
Philip Batterham
Ben Beaglehole
Xavier Benarous
Daniel Bennett
Nicole Benson
Alberto Benussi
Ilaria Benzi
Tomi Bergström
Tekola Bethlehem
Amritha Bhat
Sarmishtha Bhattacharyya
Vishal Bhavsar
Kamaldeep Bhui
Lucy Biddle
Jo Billings
Mengesha Biresaw
Mengesha Birkie
Jonathan Bisson
Istvan Bitter
Donald Black
Julio Bobes
Joseph Boden
Harm Boer
Lana Bojanić
Cailin Boland
Eva-Maria Bonin
Michel Botbol
Helen Bould
Jane Boydell
Bethany Brand
Christian Brandt
Konrad Bresin
Erica Breuer
Sofia Brissos
Jillian Broadbear
Paola Bucci
Alec Buchanan
Dejan Budimirovic
Karen Bunning
Wendy Burn
Alistair Burns
Lauren Burns
Walter Busuttil
Niels Buus
Na Cai
Jane Callaghan
Daniel M. Campagne
Rudolf Cardinal
Roxana Cardos
Gabrielle Carlson
Cecilia Casetta
Carl Castro
Nandini Chakraborty
Samuel Chamberlain
Fiona Charlson
Nasim Chaudhry
Imran Chaudhry
Qingwei Chen
Astrid Chevance
Shing Wan Choi
Stephen Clift
Lucie Cluver
Rose Collard
Pamela Collins
John Cookson
Fiammetta Cosci
Tiago Costa
Ken Courtenay
Darren Courtney
Lesley Cousins
Tom Craig
Ioana Cristea
Lauren Cross
Matthew Croucher
Marie Crowe
Maarten Cuypers
Armando D'Agostino
Oliver Dale
Anna-Karin Danielsson
Ananta Dave
Jonathan Davidson
Jana DeVilliers
Kimberlie Dean
Olivia Dean
Wendy Dean
Jessica Dennis
Eske Derks
Paul Desan
Pushpal Desarkar
Michael Deuschle
Colin Dewar
Kirsten Dillon
Anne Doherty
Geert Dom
Jade Donaghy
Katie Douglas
Małgorzata Dragan
Fiona Duffy
Michel Dückers
Klaus Ebmeier
Kate Eggleston
Stuart Eisendrath
Timon Elmer
Whiskey Eromona
Javier Escobar
Tatjana Ewais
Lisa Eyler
Hanan Ez elarab
Neus Falgas
Peter Falkai
Andrew Farmery
Joanne Farrow
Sandra Feodor Nilsson
Damián Fernández-Costa
Lucrezia Ferrante
Federica Ferrari
Mudasir Firdosi
Luciana Fonseca
Tamsin Ford
David Foreman
Andrew Forrester
Karen Fortuna
Phillipe Fossati
Sophia Frangou
Emma Fransson
Mark Freestone
Dawn Freshwater
Miquel Angel Fullana
Eva Gal
Peter Gallagher
Satheesh Kumar Gangadharan
Emily Garman
Elena Garralda
Christiane Gasse
Chiara Gastaldon
Raluca Georgescu
Domenico Giacco
María Estela Giardina
Rachel Gibbons
Dino Gibertoni
Nadeem Gire
Vivette Glover
Paul Glue
Guy Goodwin
Christopher Gordon
Ken Goss
David Gratzer
Michael Grunebaum
David Gunnell
Peter Haddad
Michael Hadjulis
Catherine Haighton
John Haltigan
Jeffrey Hankey
Ketil Hanssen-Bauer
Raveen Hanwella
Judith Harbertson
Catherine Harmer
Joyce Harrison
Judith Harrison
Alkomiet Hasan
Angela Hassiotis
Keith Hawton
Tim Healing
Claire Henderson
Chantal Henry
Jennifer Hensel
Sarah Herzog
Phillip Hessel
Walter Hewer, Prof.
Diego Hidalgo-Mazzei
Julie Highfield
Alannah Hillmer
Lindsey Hines
Paul Hoff
Matthew Hotopf
Louise Howard
Yueqin Huang
Christy Hui
Rachael Hunter
M Omair Husain
Muhammad Husain
Nusrat Husain
Jane Ireland
Jeremy Isaacs
Anupama Iyer
Ramune Jacobsen
Nicholas Jacobson
Sneha Jadhav
Haitham Jahrami
Adrian James
Carol Janney
Sameer Jauhar
Rachel Jenkins
Caroline Johnson
Roland Jones
Edgar Jones
Jessica Jones Nielsen
Jenny Jordan
Martin Jorgensen
Eileen Joyce
Mario Juruena
Bonnie Kaiser
Jeanine Kamphuis
Andres Kanner
Sujita Kar
Karen-Inge Karstoft
Tyler Kaster
Cornelius Katona
Aristeidis Katsanos
Kenneth Kaufman
Charles Kellner
Charles Kelly
Chris Kemp
Patrick Kempermann
Harry Kennedy
Michael Kerr
Hanna Kienzler
Helen Killaspy
Steve Kisely
Jan Kizilhan
Stefan Kloiber
Günter Klug
Martin Knapp
Manami Kodaka
Brandon Kohrt
Gerda Kuijper
Jayashri Kulkarni
Ashok Kumar-Jainer
Paul Kurdyak
W Curt LaFrance Jr
Cameron Lacey
Nicolai Ladegaard
Femke Lamers
Gary Lamph
Matthew Large
Mary Larkin
Celine Larkin
Miriam Larsen-Barr
Emily Lattie
Richard Laugharne
Thomas Laursen
Stephen Lawrie
Mary Leamy
Edouard Leaune
Youjin Lee
William Lee
Belinda Lennox
Peter Lepping
Yafit Levin
Robert Levitan
Kathryn Lewandowski
Glyn Lewis
Stephanie Lewis
Roberto Lewis-Fernandez
Yi Li
Luming Li
Haiqun Lin
Dennis Michael Linder
Gill Livingston
Carmen Lopez
Kate Lovero
Lin Lu
Fengmei Lu
Todd Lubart
Nagendra Luitel
Jason Luty
Sean Lynch
Ashley Lütticke
Nombulelo Madala-Witbooi
Rajneesh Mahajan
Tariq Mahmood
Gin Malhi
Almaz Mamaru
Rachel Marchant
Suzanne Mares
Joanna Martin
Joanna Maselko
Nora Mat Zin
Lorenzo Mazzarini
Orla McBride
Jane McCarthy
Catherine McHugh
Andrew McKechanie
Martin McKee
Jason Mcintyre
Nicola Meda
Alan Meehan
Eline Meijer
Werkua Mekonnen
Clare Melvin
Emily Mendenhall
Lynn Michalopoulos
Diana Miconi
Jouko Miettunen
Clara Miguel
Barbara Milrod
VIjay Mittal
Omer Moghraby
Andrew Molodynski
Emmanuel Monfort
Cecilia Montiel
Gabriela Moreno
Richard Morriss
Emma Morton
Marco Mula
Roger Mulder
Dominic Murphy
Jamie Murphy
Esther Murray
Katherine Musliner
David Ndetei
Naresh Nebhinani
Diana Nechita
Giles Newton-Howes
Dasha Nicholls
Timothy Nicholoson
Alexander Nissen
Evangelos Ntontis
David O' Regan
Aileen O'Brien
Anthony O'Brien
Rory O'Connor
Siobhan O'Dwyer
David O' Kai
Kristine Olson
Vasiliki Orgeta
Michael Ostacher
Edoardo Ostinelli
Clare Pain
Yuval Palgi
A-La Park
Adam Perkins
Anne-Cecile Petit
Eva Petkova
Theodore Petti
Thomas Phillips
Mariana Pinto da Costa
Alexandra Pitman
Michael Pluess
Thomas Pollak
Richard Porter
Adam Powell
Vibhore Prasad
Annabel Price
Kiran Purandare
Yann Quide
Leah Quinlivan
Inti Qurashi
Andrea Raballo
Ravi Ramasamy
Abdul Raoof
Justin D. Rasmussen
Elena Ratschen
Ambiga Ravi
Sarah Reeve
Jason Reichenberg
Lennart Reifels
Joe Reilly
Wagner Ribeiro
Katharine Rimes
Jonathan Rogers
Borja Romero-Gonzalez
Jan Rondeel
Larissa Rossen
Ashok Roy
Paola Rucci
Torleif Ruud
Carole Samango-Sprouse
Martha N. Sajatovic
Cynthia Salorio
Jay Salpekar
Musa Sami
Gabriele Sani
Manel Santiñà
Mayra Sapniol
Amir Sariaslan
Jerome Sarris
Alexander Sartorius
Farzan Sasangohar
Senthil Kumaran Satyanarayanan
Robert Schoevers
Jean-Paul Selten
Nick Sevdalis
Arieh Shalev
Farshid Shamsaei
Rohit Shankar
Pratap Sharan
Deborah Sharp
Marie-Louise Sharp
James Shearer
Rory Sheehan
Bart Sheehan
Christine Sheppard
Mark Shevlin
Tian-Mei Si
Najma Siddiqi
Robert Sigstrom
Derrick Silove
Cristiane Silvestre de Paula
Julia Sinclair
Om Prakash Singh
Mark Sinyor
Dan Siskind
Bonnie Siu
Ashraf Skagee
Renee Smith
Daniel Smith
Manit Srisurapanont
Corbin J Standley
Stephen Stansfeld
Iris M Steine
Joanna Steinglass
Lucy Stephenson
Robert Stewart
Paul Stokes
John Stone
James Stone
Clifford Stott
Rebecca Strawbridge
Jing Sui
Tony Sun
Jingsong Tang
Sandila Tanveer
Alvin Kuowei Tay
Maria Cristina TV Teixeira
Su-Gwan Tham
Michael Thase
Alex Thomson
Graham Thornicroft
Alix Timko
Leonardo Tondo
Paul Toner
John Torous
Ivan Torres
Derek Tracy
Wolfgang Trapp
Samuel Tromans
Ruby Tsang
Helena Tuomainen
Peter Tyrer
Bryan Tysinger
Linda Valeri
Annemarie Van Elburg
Rob van den Brink
Eva van der Ploeg
Anna Van Meter
Astrid Vellinga
Peter Ventevogel
Jolien Veraart
Norma Verdolini
Simone Vigod
Lakshmi Vijayakumar
Juliet Wakefield
Robert Walker
Melanie Wall
Sonya Wallbank
Glenn Waller
Jijun Wang
Ya Wang
Hong-xing Wang
Wayne Warburton
Danuta Wasserman
Simon Wessely
Richard Whale
Diana Whalen
Ross White
Randall White
Richard Whittington
Richard Williams
Jill Williams
Jonathan Wilson
Jean Wolstencroft
Hoffman Yaakov
Sosei Yamaguchi
Min Yang
Philip Yanos
Allan Young
Simon Young
Ti-Fei Yuan
Weihua Yue
Roland Zahn
Clement Zai
Caroline Zangani
Yosra Zgueb
Xiao-mei Zhong
Liang Zhou
Carolina Ziebold
Stephan Zipfel
Tiago Zortea

